# Racial Differences in 30-Day Reintubation After Head and Neck Surgery

**DOI:** 10.7759/cureus.35280

**Published:** 2023-02-21

**Authors:** Brittany N Burton, Pelle V Wall, Danny Le, Adam J Milam, Rodney A Gabriel

**Affiliations:** 1 Anesthesiology, David Geffen School of Medicine, University of California, Los Angeles, USA; 2 Anesthesiology, San Diego School of Medicine, University of California, La Jolla, USA; 3 Anesthesiology, Mayo Clinic, Phoenix, USA

**Keywords:** reintubation, postoperative complicaiton, postoperative outcomes, otolaryngology-head and neck surgery, race inequities

## Abstract

Background

This study aimed to examine the association of race and ethnicity with 30-day unplanned reintubation following head and neck surgery.

Methodology

A retrospective analysis of head and neck surgery patients aged greater than or equal to 18 years was extracted from the American College of Surgeons National Surgical Quality Improvement Program database from 2015 to 2020. Patient demographics, comorbidities, and 30-day reintubation were included in the analysis. Pearson’s chi-square and independent samples t-test were used to compare reintubation cohorts. Multivariable logistic regression was used to identify the association of race and ethnicity with 30-day reintubation.

Results

Of the total 108,442 head and neck surgery cases included, 74.9% of patients were non-Hispanic White, 17.3% were non-Hispanic Black, and 7.7% were Hispanic. The overall 30-day reintubation rate was 0.33%. After adjusting for age, body mass index, sex, and comorbidities, non-Hispanic Black patients had increased 30-day reintubation compared to non-Hispanic White patients (odds ratio [OR] = 2.14, 95% confidence interval [CI] 1.70-2.69, and *P *< 0.0001). There was no difference in 30-day reintubation for Hispanic patients compared to non-Hispanic White patients (OR = 1.08, 95% CI 0.67-1.65, and *P *= 0.747).

Conclusions

This analysis showed that non-Hispanic Black patients disproportionately had higher odds of 30-day reintubation following head and neck surgery. Hispanic ethnicity was not associated with increased odds of 30-day reintubation. More studies are needed to investigate the reasons for these racial differences.

## Introduction

In the United States, advancements in head and neck surgery have improved morbidity and mortality rates to as low as 5.65% and 2.98%, respectively; however, preventable postoperative complications continue to occur [1]. Involvement of the airway and the complexity of head and neck surgery cases present many potential postoperative complications [2,3]. Pulmonary complications following head and neck surgery may necessitate unplanned postoperative reintubations.

Unplanned reintubation is a significant postoperative adverse event, as it is associated with increased postoperative pneumonia, tracheostomy, length of hospital stay, mortality, and greater financial burden on the hospital and patients [4,5]. In a case-control study by Chen et al. involving 123,068 surgical patients, risk factors associated with reintubation included age greater than 65 years, increased American Society of Anesthesiologists (ASA) physical status classification, high fluid volume status, and head and neck or thoracic surgery [6].

Among the risk factors for poor surgical outcomes, race and ethnicity have been highlighted [7-9]. Little information exists in the literature regarding the association of race and ethnicity on reintubation rates across the common head and neck surgeries. Therefore, we aimed to assess the association of race and ethnicity with 30-day postoperative unplanned reintubation following head and neck surgery using the ACS National Surgical Quality Improvement Project (ACS NSQIP). We hypothesized that there would be racial differences in this important postoperative complication.

## Materials and methods

The ACS NSQIP 2015 to 2020 database was used for this cohort study [10]. ACS NSQIP is a surgical outcomes program used to improve perioperative healthcare. This study was exempt from institutional review board approval and adheres to the Strengthening the Reporting of Observational Studies in Epidemiology (STROBE) Enhancing the Quality and Transparency of Health Research (EQUATOR) guidelines. All cases were aged greater than or equal to 18 years.

We extracted cases from ACS NSQIP Participant Use File 2015 to 2020, with a primary surgery defined with Current Procedural Terminology (CPT) code as (1) parathyroidectomy (60500, 60502, 60505, and 60512), (2) thyroidectomy (60210, 60212, 60220, 60225, 60240, 60252, 60254, 60260, 60270, and 60271), (3) glossectomy (41135, 41140, 41145, and 41155), and (4) parotid tumor excision (42420, 42425, and 42426). These surgical procedures were included due to their potential risk for serious pulmonary complications that would require reintubation. Parathyroidectomy and thyroidectomy are associated with cervical hematoma or vocal cord paralysis, and glossectomy and parotidectomy lead to significant airway edema. These complications may lead to life-threatening airway compromise and necessitate postoperative reintubation.

**Figure 1:**
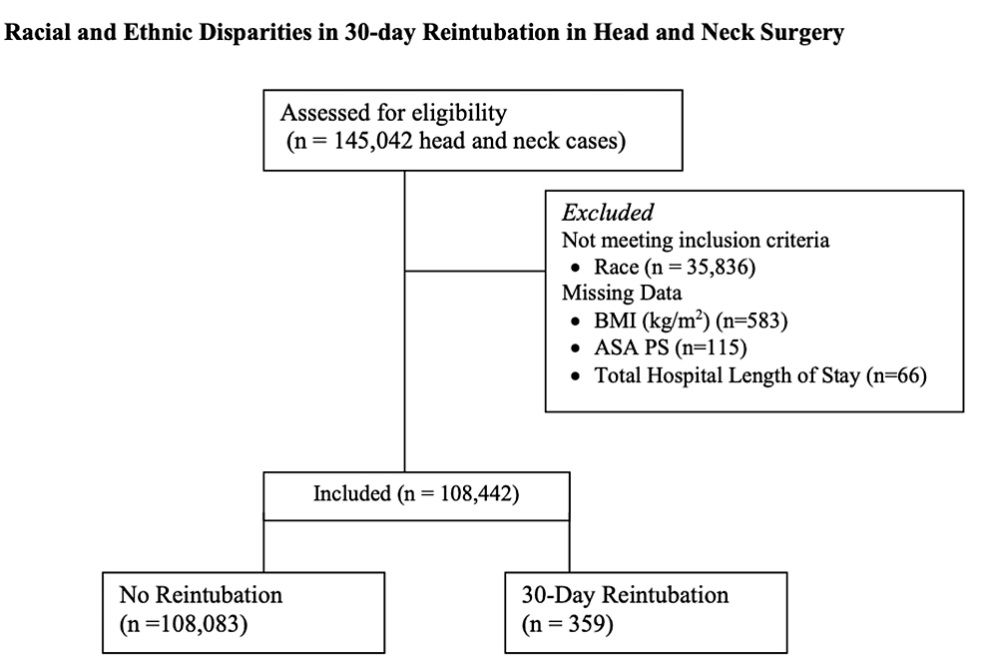
Flow diagram of inclusion and exclusion criteria. Figure credits: Brittany N. Burton. BMI, body mass index; ASA, American Society of Anesthesiologist

Self-identified race and ethnicity was the primary independent variable. The three racial cohorts were (1) non-Hispanic White, (2) non-Hispanic Black, and (3) Hispanic. The primary outcome was unplanned 30-day postoperative reintubation. ACS NSQIP defined unplanned reintubation as an event where a patient required placement of an endotracheal tube or other similar breathing device and mechanical ventilation within 30 days following surgery, which was not intended or planned [11]. The following variables were included in the multivariable logistic regression analysis based on their importance in perioperative medicine: age, body mass index, diabetes mellitus, active smoking history, congestive heart failure, metastatic cancer, hypertension, bleeding disorder, chronic obstructive pulmonary disease, cerebrovascular accident, and ASA class. Exclusion criteria included race/ethnicity not self-identified as non-Hispanic White, non-Hispanic Black, or Hispanic; missing data for race/ethnicity; body mass index; ASA class; and length of hospital stay (Figure 1).

Statistical analysis

This was a retrospective cohort study, in which the cohorts were patients that did not require reintubation versus patients who did require an unplanned reintubation within 30 days after their surgery. Independent samples t-test and Pearson’s chi-square tests were used to compare reintubation cohorts. We used multivariable logistic regression to model the association of race and ethnicity with 30-day reintubation. Covariates included in the multivariable model were age, body mass index, diabetes mellitus, smoking history, congestive heart failure, metastatic cancer, hypertension, bleeding disorder, chronic obstructive pulmonary disease, cerebrovascular accident, and ASA score. The odds ratio (OR) and 95% confidence interval (CI) were presented and a *P *< 0.05 was considered statistically significant for outcome measures. R (version 4.0.3, R Foundation for Statistical Computing, Vienna, Austria; 20 October, 2020) was the statistical computing platform used to perform statistics.

## Results

There were 145,042 head and neck cases identified in ACS NSQIP from 2015 to 2020. After exclusion criteria were considered (Figure 1), the final sample size was 108,442. Among the study population, the rate of 30-day reintubation was 0.33% (Table 1). The rates of no reintubation versus 30-day reintubation among racial cohorts were the following: for non-Hispanic White 75% versus 57.7%, non-Hispanic Black 17.3% versus 36.5%, and Hispanic 7.8% versus 5.8%, *P *< 0.001. The rate of 30-day reintubation was significantly higher for older patients and patients with disseminated cancer, diabetes mellitus, hypertension, chronic obstructive pulmonary disease, cerebrovascular accident, and active smokers and patients with higher ASA status (*P *< 0.001).

**Table 1:** Patient characteristics. IQR, interquartile range

Perioperative factors	No reintubation, *n* (%)	30-day reintubation, *n* (%)	*P*-value
Sample size	108,083 (99.6)	359 (0.33)	
Race			<0.001
non-Hispanic White	81,030 (75.0)	207 (57.7)	
non-Hispanic Black	18,659 (17.3)	131 (36.5)	
Hispanic	8,394 (7.8)	21 (5.8)	
Body mass index (kg/m^2^), median (IQR)	29.93 (25.69-35.33)	30.50 (26.19-37.20)	0.071
Age (year), median (IQR)	60 (46-73)	65 (53-74)	<0.001
Disseminated cancer	1,043 (1.0)	15 (4.2)	<0.001
Diabetes mellitus			<0.001
No	91,773 (84.9)	261 (72.7)	
Noninsulin dependent	11,111 (10.3)	54 (15.0)	
Insulin dependent	5,199 (4.8)	44 (12.3)	
Tobacco smoke	14,312 (13.2)	81 (22.6)	<0.001
Hypertension	49,188 (45.5)	249 (69.4)	<0.001
Bleeding disorder	1,561 (1.4)	8 (2.2)	0.307
Chronic obstructive pulmonary disease	3,020 (2.8)	34 (9.5)	<0.001
Cerebrovascular accident	39 (0.0)	7 (1.9)	<0.001
American Society of Anesthesiology Class			<0.001
No disturbance	4,175 (3.9)	3 (0.8)	
Mild disturbance	59,685 (55.2)	92 (25.6)	
Severe disturbance	41,673 (38.6)	213 (59.3)	
Life threatening	2,546 (2.4)	51 (14.2)	
Moribund	4 (0.0)	0 (0.0)	

**Figure 2:**
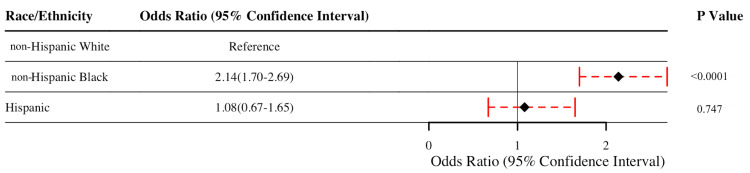
Forest plot of race and ethnicity with 30-day reintubation. Figure credits: Brittany N. Burton.

On multivariable analysis, non-Hispanic Black patients (OR = 2.14, 95% CI 1.70-2.69, and *P *< 0.0001) had significantly higher adjusted odds of reintubation compared to non-Hispanic White patients. There was no difference in the adjusted odds of reintubation for Hispanic compared to non-Hispanic White patients (OR = 1.08, 95% CI 0.67-1.65, and *P *= 0.747; Figure 2).

 

## Discussion

This study demonstrated that non-Hispanic Black patients had higher adjusted odds of undergoing 30-day reintubation compared with non-Hispanic Whites. Thirty-day postoperative reintubation was associated with many variables, including disseminated cancer, diabetes mellitus, hypertension, chronic obstructive pulmonary disease, cerebrovascular accident, active smokers, and patients with higher ASA status. Taken together, our studies provide observational evidence that there may be differences in racial/ethnic groups for postoperative outcomes following head and neck surgery. It is, however, important to note that the results of this study do not prove the existence and/or reasons for healthcare disparities in this surgical population. Rather, these results should be used to drive future hypotheses when designing research studies aimed at identifying the presence and causes of racial disparities in perioperative medicine.

Head and neck surgery has previously been identified as a risk factor for unplanned postoperative reintubation, with notable racial differences in outcomes in the pediatric literature [6,12,13]. One study showed that Black children had higher odds of perioperative pulmonary complications after otolaryngology surgery, including twofold higher odds for unplanned reintubation [14]. Another study found higher rates of respiratory events including laryngo/bronchospasm, pneumonia, pulmonary edema, intubation, prolonged intubation, and ventilation in Black children after tonsillectomy compared with children of other races [15]. Furthermore, Malyavko et al. found that children from underrepresented minority groups had higher odds of undergoing unplanned reintubation compared with White children following surgical treatment of hip dysplasia [16].

The potential reasons for racial and ethnic differences in postoperative complications are multifactorial. One potential reason could be related to the data represented in NSQIP. While it captures over 700 healthcare institutions, it is not necessarily representative of the entire United States. Furthermore, NSQIP does not provide data on which institutions each patient was treated; thus, analysis was done on the entire collective population and, consequently, did not control for individual geographic/hospital practices. Therefore, it was not possible to determine if there were racial differences within each institution.

Other possible reasons for the observed racial differences in unplanned reintubations could be related to the quality of care and provider experience in areas with higher proportion of minorities. For example, surgery for Grave’s disease was associated with higher risk for complications when performed by surgeons with less experience [17]. In a study from 2007, it was reported that majority of Hispanic and Black patients had thyroid surgery by the lowest-volume surgeons compared to White patients [18]. In their study, Noureldine et al. reported a longer length of hospital stay, increased postoperative complications, and increased costs for Black patients compared to White patients after thyroid and parathyroid patients [19]. In that study, it was reported that African Americans had less access to intermediate and high-volume surgeons [16].

Delayed access or presentation to quality healthcare providers may constitute another factor in our observed outcomes. Kuo et al. reported that Black patients experienced longer times to surgical referral for benign thyroid disease, presented with significantly larger thyroid glands, and displayed more compressive symptoms and dysphagia than White patients in a single-institution study. These patients also experienced significantly higher rates of postoperative reintubation [20]. Black patients presenting with more advanced thyroid disease and requiring urgent and emergent surgery has also been described elsewhere and could potentially contribute to the higher rates of postoperative intubation seen in this study [18].

In addition to presenting later in the course of the surgically indicated disease, racial minorities and Black patients in particular may have more severe comorbidities that affect postoperative complications. For instance, Black patients have been shown to have more severe obstructive sleep apnea at diagnosis and poor treatment outcomes compared to other races [21]. More severe obstructive sleep apnea has been shown to confer increased risk of postoperative respiratory complications including reintubation and an increased association with preoperative diabetes mellitus and hypertension [22]. While our analysis attempted to control for certain comorbidities in comparing unplanned reintubation rates, the NSQIP database did not report on the severity of such conditions; as such, there may be systematic disparities in comorbidity severity for which we could not account.

The root causes underpinning the aforementioned racial disparities in access to high-volume surgeons, delays in medical care, and comorbidity severity are likely multifactorial. Socioeconomic status is likely one element, as it informs access to healthcare, environmental exposure, and health behavior [23]. Distrust in the medical system due to historic and ongoing discriminatory practices could also contribute. In one study from 2003, non-Hispanic Black patients reported lower levels of trust in their physicians than non-Hispanic White patients and more concern about potential harmful experimentation in hospitals [24]. One study from 2019 found that 22% of Black patients avoided seeking medical care for themselves or a family member due to anticipated discrimination [25]. Lack of therapeutic alliances between practitioners and patients may also contribute to the milieu responsible for racial health disparities. In a 2003 study, poor interpersonal skills were reported in physician interactions with Black and Hispanic patients, with physicians more likely to provide these patients will less information [26].

This study adds to the growing body of literature showing racial differences in postoperative outcomes, which are likely caused by a complex of contributing factors. Healthcare professionals should be aware of historical and ongoing shortcomings in communication and therapeutic alliances and seek to understand the patient’s concerns when possible. Proactively gaining the trust of all patients and cultivating an awareness of any implicit and unconscious biases that may hamper clinical relationships and contribute to suboptimal care.

While this study has many strengths, it also has limitations. The data for this analysis was obtained from the NSQIP database and therefore possesses limitations inherent to large national databases, including relying on accurate coding and complete documentation of information. As data is collected from large academic institutions, the external validity of our findings for community and public hospitals is limited. The database also does not provide information on patient socioeconomic or insurance status, which, as previously discussed, are significant contributors to health outcomes. Additionally, the severity of comorbidities that may have contributed to differences in reintubation rates between races could not be assessed. Race was self-reported, and patients who did not select a race or who indicated a race other than non-Hispanic Black, non-Hispanic White, or Hispanic were not included. These exclusions reduce our sample size and decrease the power of the study.

## Conclusions

This study presents a large-scale analysis of reintubation rates after common head and neck surgeries for non-Hispanic Black, non-Hispanic White, and Hispanic patients. The findings from the present analysis demonstrate ongoing racial disparities in postoperative outcomes with significant associated morbidity. While the etiology of these findings is complex, providers and health systems can take proactive steps to mitigate contributing factors within their control. Such actions include acknowledging and addressing implicit biases that may undermine communication and therapeutic alliances with patients.

## References

[1] Lin HW, Bhattacharyya N (2012). Contemporary assessment of medical morbidity and mortality in head and neck surgery. Otolaryngol Head Neck Surg.

[2] McMahon JD, MacIver C, Smith M (2013). Postoperative complications after major head and neck surgery with free flap repair - prevalence, patterns, and determinants: a prospective cohort study. Br J Oral Maxillofac Surg.

[3] Ong SK, Morton RP, Kolbe J, Whitlock RM, McIvor NP (2004). Pulmonary complications following major head and neck surgery with tracheostomy: a prospective, randomized, controlled trial of prophylactic antibiotics. Arch Otolaryngol Head Neck Surg.

[4] Burton BN, Abudu B, Bhat P, Gabriel RA, Schmidt UH (2019). Thirty-day unplanned reintubation following pleurodesis: a retrospective national registry analysis. J Cardiothorac Vasc Anesth.

[5] Tillquist MN, Gabriel RA, Dutton RP, Urman RD (2016). Incidence and risk factors for early postoperative reintubations. J Clin Anesth.

[6] Chen S, Zhang Y, Che L, Shen L, Huang Y (2021). Risk factors for unplanned reintubation caused by acute airway compromise after general anesthesia: a case-control study. BMC Anesthesiol.

[7] Liu JC, Egleston B, Blackman E, Ragin C (2022). Racial survival disparities in head and neck cancer clinical trials. J Natl Cancer Inst.

[8] Moon PK, Qian ZJ, Noel JE (2022). Sociodemographic disparities in the diagnostic management of pediatric thyroid nodules. JAMA Otolaryngol Head Neck Surg.

[9] Goljo E, Parasher AK, Iloreta AM, Shrivastava R, Govindaraj S (2016). Racial, ethnic, and socioeconomic disparities in pituitary surgery outcomes. Laryngoscope.

[10] Raval MV, Pawlik TM (2018). Practical guide to surgical data sets: National Surgical Quality Improvement Program (NSQIP) and pediatric NSQIP. JAMA Surg.

[11] (2022). The American College of Surgeons National Surgical Quality Improvement Program. https://www.facs.org/quality-programs/acs-nsqip..

[12] Lin HT, Ting PC, Chang WY, Yang MW, Chang CJ, Chou AH (2013). Predictive risk index and prognosis of postoperative reintubation after planned extubation during general anesthesia: a single-center retrospective case-controlled study in Taiwan from 2005 to 2009. Acta Anaesthesiol Taiwan.

[13] Ting PC, Chou AH, Yang MW, Ho AC, Chang CJ, Chang SC (2010). Postoperative reintubation after planned extubation: a review of 137,866 general anesthetics from 2005 to 2007 in a Medical Center of Taiwan. Acta Anaesthesiol Taiwan.

[14] Sivak E, Mpody C, Willer BL, Tobias J, Nafiu OO (2021). Race and major pulmonary complications following inpatient pediatric otolaryngology surgery. Paediatr Anaesth.

[15] Kou YF, Sakai M, Shah GB, Mitchell RB, Johnson RF (2019). Postoperative respiratory complications and racial disparities following inpatient pediatric tonsillectomy: a cross-sectional study. Laryngoscope.

[16] Malyavko A, Quan T, Howard PG, Recarey M, Manzi JE, Tabaie S (2022). Racial disparities in postoperative outcomes following operative management of pediatric developmental dysplasia of the hip. J Pediatr Orthop.

[17] Kandil E, Noureldine SI, Abbas A, Tufano RP (2013). The impact of surgical volume on patient outcomes following thyroid surgery. Surgery.

[18] Sosa JA, Mehta PJ, Wang TS, Yeo HL, Roman SA (2007). Racial disparities in clinical and economic outcomes from thyroidectomy. Ann Surg.

[19] Noureldine SI, Abbas A, Tufano RP, Srivastav S, Slakey DP, Friedlander P, Kandil E (2014). The impact of surgical volume on racial disparity in thyroid and parathyroid surgery. Ann Surg Oncol.

[20] Kuo LE, Simmons KD, Wachtel H, Zaheer S, Karakousis GC, Fraker DL, Kelz RR (2016). Racial disparities in initial presentation of benign thyroid disease for resection. Ann Surg Oncol.

[21] Dudley KA, Patel SR (2016). Disparities and genetic risk factors in obstructive sleep apnea. Sleep Med.

[22] Munish M, Sharma V, Yarussi KM, Sifain A, Porhomayon J, Nader N (2012). The use of practice guidelines by the American Society of Anesthesiologists for the identification of surgical patients at high risk of sleep apnea. Chron Respir Dis.

[23] Adler NE, Newman K (2002). Socioeconomic disparities in health: pathways and policies. Health Aff (Millwood).

[24] Boulware LE, Cooper LA, Ratner LE, LaVeist TA, Powe NR (2003). Race and trust in the health care system. Public Health Rep.

[25] Bleich SN, Findling MG, Casey LS (2019). Discrimination in the United States: experiences of black Americans. Health Serv Res.

[26] Ashton CM, Haidet P, Paterniti DA (2003). Racial and ethnic disparities in the use of health services: bias, preferences, or poor communication?. J Gen Intern Med.

